# A COVID-19 model incorporating variants, vaccination, waning immunity, and population behavior

**DOI:** 10.1038/s41598-022-24967-z

**Published:** 2022-11-27

**Authors:** Zachary LaJoie, Thomas Usherwood, Shailen Sampath, Vikas Srivastava

**Affiliations:** 1 School of Engineering, Brown University, Providence, RI 02912 USA; 2 Center for Biomedical Engineering, Brown University, Providence, RI 02912 USA

**Keywords:** Viral infection, Computational models, Biomedical engineering

## Abstract

Vaccines for COVID-19 have allowed countries to combat the spread of the disease. However, new variants have resulted in significant spikes in cases and raised severe health and economic concerns. We present a COVID-19 model to predict coupled effects of vaccine multiple-dose roll-out strategies, vaccine efficacy, waning immunity, population level of caution, sense of safety, under-reporting of cases, and highly prevalent variants such as the Delta (B.1.617.2) and Omicron (B.1.1.529). The modeling framework can incorporate new variants as they emerge to give critical insights into the new cases and guide public policy decision-making concerning vaccine roll-outs and reopening strategies. The model is shown to recreate the history of COVID-19 for five countries (Germany, India, Japan, South Africa, and the United States). Parameters for crucial aspects of the pandemic, such as population behavior, new variants, vaccination, and waning immunity, can be adjusted to predict pandemic scenarios. The model was used to conduct trend analysis to simulate pandemic dynamics taking into account the societal level of caution, societal sense of safety, and the proportions of individuals vaccinated with first, second, and booster doses. We used the results of serological testing studies to estimate the actual number of cases across countries. The model allows quantification of otherwise hard to quantify aspects such as the infectious power of variants and the effectiveness of government mandates and population behavior. Some example cases are presented by investigating the competitive nature of COVID variants and the effect of different vaccine distribution strategies between immunity groups.

## Introduction

Severe acute respiratory syndrome coronavirus 2 (SARS-CoV-2), the virus that causes coronavirus disease 2019 (COVID-19), was first detected in Wuhan, China in December of 2019. Since its initial detection, the virus has resulted in a global pandemic of COVID-19 with over 500 million cases and 6 million deaths world-wide^1^. In response, most countries had lock downs that negatively impacted local and global economies^2^. The recent developments of COVID vaccines, such as BNT162b2 (Pfizer-BioNTech), mRNA-1273 (Moderna), and ChAdOx1 (AstraZeneca), have helped in curbing the spread of the disease^3,4^. Accelerated vaccine roll-outs have been shown to significantly reduce the number of cases within countries^5^. As of March of 2022, there are currently 12 vaccines approved for use and 38 vaccines in the final stages of testing^6^. Additionally, there have been 5 billion individuals who have been vaccinated with at least one dose world-wide^7^.

However, as promising new vaccines have emerged, variants of concern (VOC) bring new uncertainties and complicate curbing of the disease. VOCs, as defined by the United States Center for Disease Control and Prevention, are more transmissible versions of SARS-CoV-2; they tend to be more severe and result in a significant reduction in vaccine effectiveness^8^. Several VOCs have emerged since December 2020. The Alpha variant (B.1.1.7) was first detected in the United Kingdom and had a 50% increased transmission, soon after the Beta variant (B.1.351) was documented in South Africa. The Gamma variant (P.1) was first detected in Brazil^9^, and the Delta variant (B.1.167.2) was first identified in India and classified on May 11th, 2021^10^. The Omicron variant (B.1.1.529) was first detected in South Africa and Botswana in 2021, and this VOC is even more transmissible than Delta, however, it is less severe than previous VOCs^11^. These variants have contributed to recent large new COVID case surges across countries. When the Delta variant became the dominant VOC around the world, highly vaccinated countries saw significant increases in cases^12^. Delta was more dangerous than previous variants, and vaccines are less effective in protecting against it. Getting one dose of either BNT162b2 (Pfizer-BioNTech) or ChAdOx1 (AstraZeneca) confers 25–36% immunity against the Delta compared to 45–52% immunity against the Alpha VOC^12^. Receiving two doses was also shown to confer less immunity against the Delta variant^12^. Lower immunity, combined with the fact that Delta was more transmissible than previous VOCs^8^ (due to the presence of a mutation in the Delta SARS-CoV-2 spike protein’s receptor-binding domain^13^) led to the immense increases in infectious cases we observed around the world in 2021. In December of 2021, the Omicron variant led to even more significant surges in cases across the globe. Omicron has been found to have 33 mutations in its spike protein compared to the wild type strain^14^. These large mutations that have been seen across various VOCs require that countries worldwide reconsider the best way to roll out vaccines considering vaccines become less effective and society reopens (more infectious interactions) with time.

As of Spring 2022, most current vaccines require two doses that are recommended to be given 3 to 4 weeks apart on average, require storage at chilled temperatures, and have a limited shelf life^15^. Currently, getting an additional one or two booster shots is necessary to retain high levels of protection^16^. Navigating these requirements has been difficult globally. For countries that are not producing vaccines and rely on importing doses^17^, the vaccine availability has been limited, and a large number of people are put at risk. As a result, many countries with lower supply levels have considered using their vaccine supply to give as many first doses as they can, which still confer reasonable immunity and delay the second dose’s time compared to the recommended time frame. This vaccination strategy of prioritizing first doses has had success for other outbreaks in the past in managing delays in vaccination supplies. During a cholera outbreak in Zambia, a single-dose vaccine campaign was successfully used to help get doses to the majority of individuals even though two vaccines were recommended^18^. In 2016, one-fifth of the recommended dosage of the yellow fever vaccine was used to successfully respond to a yellow fever outbreak in Angola, Uganda, and the Democratic Republic of the Congo^19^. While most countries have prioritized using two dose deployment^15^ in accordance with guidelines for the COVID-19 vaccine by the World Health Organization^20^, many countries decided to prioritize giving first doses to specific segments of the population or most of the population first when they were low on vaccine supplies. In Canada, a 4-months gap between doses was used, with the country prioritizing giving doses to the elderly and those most at risk first^21^. The United Kingdom also allowed for a 12 week delay in the second dose for both the BNT162b2 (Pfizer-BioNTech) and mRNA-1273 (Moderna) vaccines^22^. India also created a long gap of three to four months between doses^23^. The waning of vaccine protection has further created a serious concern^24^. Now that boosters are recommended, a dichotomy has been made between countries still trying to get the majority of their country vaccinated and countries trying to distribute additional booster shots.

Since the onset of COVID-19 many papers have investigated modeling the progression of the pandemic using modified differential equation compartment models that have compartments for susceptible, infectious, and recovered individuals, referred to as SIR models^25–34^. For COVID-19, various factors representing more realistic scenarios can be incorporated in the SIR models to improve predictions. Mak et al.^35^ applied a modified SEIR compartment model to take into account varying vaccine deployment strategies to predict total infections, hospitalizations, and mortality. Garcia et al.^36^ applied a model to forecast the transmission dynamics of COVID-19 in Costa Rica considering two different vaccination rate scenarios. Gonzalez-Parra et al.^37^ used a modified compartment model that incorporated latent, infective symptomatic, and symptomatic individuals to take into account VOC such as Alpha variant (B.1.1.7) to predict prevalence, hospitalizations, and deaths. However, despite numerous useful studies^38–42^, there remains a gap in modeling the coupled effects of population behavioral changes, waning immunity, vaccine roll-out strategies, and new VOCs on the progression of the pandemic.

Previously Usherwood et al.^25^ proposed a model that accounted for the population behavior and predicted infection dynamics for the original COVID-19 variant. This model was able to predict the trend of the original COVID-19 variant well but it did not consider new variants. Since then, new variants have emerged that require a model that incorporates VOCs. In this paper, we present a modeling framework that accounts for various VOCs, vaccine roll-out strategies, waning immunity, population level of caution, sense of safety, and the under-reporting of cases^43^ to replicate and predict the number of COVID-19 cases across five countries (Germany, India, Japan, South Africa, and the United States) selected to represent a diverse geographical and societal, cultural pool. We show that the model, with its computational implementation, can reasonably accurately recreate the entire history of the COVID-19 pandemic across these countries and provide a new framework to model a variety of future trends, including the contemplative studies of COVID-19 pandemic outcomes. Such modeling studies are valuable because they enable assessments of the effects of specific aspects of the pandemic, such as behavioral changes or vaccination, in isolation from other features that would affect the infection rate.

## Methods

### Modeling multiple-dose vaccination and emerging variants with a stacked model

**Figure 1:**
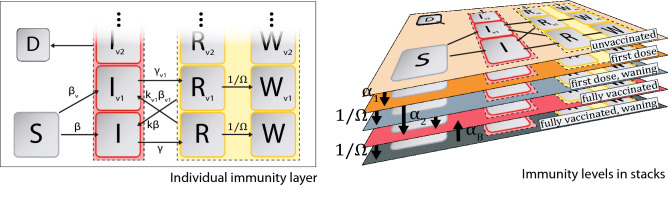
Schematic representation of proposed stacked compartment model. We propose a model that splits the population into a series of vaccination-related immunity levels and uses sub-compartments within each level to represent variants and infection-related disease states. The 2D figure (left) represents the compartment model within an immunity level, composed of a single susceptible (S) and deceased (D) compartment, as well as an infected (I), recovered (R), and waning recovered (W) compartment for each prevalent variant. The 3D figure (right) shows the entire model, where stacked immunity levels represent waning vaccination status. The dotted boxes contain several sub-compartments. Arrows representing flow from the dotted boxes in the 2D figure indicate proportional flow from each of the contained sub-compartments.

Usherwood et al.^25^ proposed a method for incorporating time-dependent population behavior changes into a SIRDV differential equation compartment model to predict original COVID-19 virus infection case trajectories for several regions of the United States. Since the publication of this work, the emergence of a large number of new virus variants, waning immunity, and a need for multiple vaccination doses has prompted the development of the new modeling framework presented in this paper. The stacked compartment framework proposed in this work is able to model the waning immunity, multiple-dose vaccinations, and new variants. The model replicates the SIRD model into the desired number of distinct immunity levels, with a fully immunized and waning level for each vaccine dosage. This results in five immunity levels/stacks: Unvaccinated (UV), First Dose (V1), First Dose Waning (V1W), Fully Vaccinated (V2), and Fully Vaccinated Waning (V2W). Each of these levels consists of a detailed compartmental model with compartments for each disease state. The model incorporates different variants by employing separate infected and recovered compartments for each variant. Waning immunity following vaccination is modeled by adding a waning immunity level for each vaccination level of the stacked compartment model. Immediately upon gaining immunity from vaccination or infection, there is flow into a compartment/layer with increased immunity; then, there is a gradual decay into the complementary waning layer with lower immunity. A similar approach is used for infection-induced immunity, where there is flow from the recovered compartment to the waning-recovered compartment, which has a greater chance for reinfection. Each layer in the model has five main compartment categories: Susceptible (S), Infected (I), Recovered (R), Waning Recovered (W), and Diseased (D), with Infected, Recovered, and Waning Recovered composed of several compartments equal to the number of variants that are being modeled. Each compartment represents a time-dependent fractional variable of the total population in each respective country, with each sub-population of the country being identifiable with a vaccination-induced immunity level (UV, V1, V1W, V2, V2W) and an infection-induced compartment within the immunity level (S, I, R, W, D). The general architecture of our model is represented in Fig. 1 and can be described by the following equations, with the parameters described in Table 1.

**Table 1:** Parameters in the model and their physical descriptions.

Parameter	Description
$\beta _v$	Transmission rate of COVID-19 variant *v*
$\alpha _1$	Rate at which people receive first vaccine dose
$\alpha _2$	Rate at which people receive second vaccine dose
$\alpha _B$	Rate at which people receive booster shots
$\eta _v^{(i)}$	Efficacy of immunity level (i) to COVID-19 variant *v*
$\gamma _v$	Recovery rate for COVID-19 variant *v*—reciprocal of infectious period
$k_v$	Relative reinfection rate for COVID-19 variant *v*—ratio of variant transmission rate before and after initial infection
$\mu _v$	Mortality rate for COVID-19 variant *v*
$\Omega$	Immunity decay rate
$d_I$	Level of caution factor-at higher values, the population will tend to increase preventative measures more quickly in response to increases in infection
$d_V$	Sense of safety factor-at higher values, the population will tend to reduce preventative measures more quickly in response to increases in vaccination. This effect counteracts the effects of level of caution.
$\beta _{v0}$	Baseline transmission rate for variant *v*, before any behavioral response to the disease

1 $$\begin{aligned} \begin{array}{l} \dfrac{dS^{(i)}}{dt}=-S^{(i)}\sum _{j}^{immunity}\sum _{v}^{variants}\Big (\beta _{v} I^{(j)}_{v}(1-\eta ^{(i)}_{v}) \Big ) \\ \dfrac{dI_v^{(i)}}{dt}=I^{(i)}_v\Big (\beta _v S^{(i)}(1-\eta _v^{(i)})-\mu _v\gamma _v-\gamma _v\Big ) \\ \dfrac{dR^{(i)}_v}{dt}=\gamma _v I_v^{(i)} - \frac{R^{(i)}_v}{\Omega } \\ \dfrac{dW^{(i)}_v}{dt}= \frac{R^{(i)}_v}{\Omega } - W^{(i)}_v\sum _{j}^{immunity}\sum _{u\ne v}^{variants}\Big (k_u\beta _{u} I_{u}^{(j)}(1-\eta ^{(i)}_{u}) \Big ) \\ \dfrac{dD^{(i)}}{dt}=\sum _{v}^{variants}\Big (\mu _v \gamma _v I_v^{(i)}\Big )\\ \end{array}. \end{aligned}$$

The transmission rate of each variant $\beta _v$ is modeled as proposed in the behavioral model by Usherwood et al.^25^. This model incorporates behavioral changes to the total infections and vaccinations as a function for the transmission rate that is dependent on the sum of all infected compartments, I, and the sum of all vaccinated levels, V.

2 $$\begin{aligned} \begin{array}{l} f_I=e^{-d_I I} \\ f_V=\dfrac{1}{f_I}+\left( 1-\dfrac{1}{f_I}\right) e^{-d_V V} \\ \beta _v=\beta _{v0} \,f_I \, f_V \end{array}. \end{aligned}$$

These equations describe the change in disease transmission as a result of behavioral response to the current number of infections and vaccinations in the population. The time dependent infectious variant transmission rate $\beta _v(t)$ is given by $\beta _v = \beta _{v0} f_I f_V$, where $\beta _{v0}$ is the population maximum infection transmission rate for a variant observed in the absence of any preventative societal measures. The expression for $f_I$ models caution in a population where individuals increase their measures of reducing transmission in response to rises in the current number of infections. A population’s tendency to take these measures in response to increases in infections is modeled by the population’s level of caution, $d_I$. The mathematical formulation is such that, when there are few infections, $I \rightarrow 0$, or when the population has a low level of caution, $d_I \rightarrow 0$, $f_I$ will approach 1 and therefore have no effect on the variant transmission rate, $\beta _v$. This represents little change toward disease safety focused behavior as a result of either a low number of infections or a low level of caution in the population. In the limiting case of very high infections and high level of caution, $f_I$ approaches 0, thereby reducing the net transmission towards 0. This scenario occurs when a population takes specific measures to significantly reduce disease transmission, such as quarantine, mask-wearing, and improved hygiene.

There is a similar but competing formulation for the population’s response to the current number of vaccinations, in which the population lessens their measures to reduce transmission as a larger proportion of the population becomes vaccinated. In the expression for $f_V$, this effect (lessening of caution due to high vaccinations) becomes negligible as the vaccinated population, V, or the population’s sense of safety, $d_V$, approaches 0. In this case, $f_V$ approaches 1 and the population’s disease transmission is determined entirely by the population’s response to infections, described above. In the limiting case of high sense of safety and high vaccination, the expression for $f_V$ approaches $\frac{1}{f_I}$, the sense of safety from vaccination negates the sense of caution from infections and the population’s disease transmission approaches its pre-pandemic level.

The net flow between immunity levels is described by first, second, and booster vaccination rates, $\alpha _1$, $\alpha _2$, and $\alpha _B$, distributed equally between compartments within the immunity level. The superscript (*i*) represents the immunity levels so that there is a set of equations of the form in Eq. (1) for each immunity level UV, V1, V1W, V2, V2W. Vaccination-induced immunity decays naturally from the first-dose and fully-vaccinated immunity levels into their respective waning immunity levels, with an equivalent form as the decay from infected to recovered compartments. Similarly, infection-induced immunity decays from the recovered to recovered waning compartment within each immunity level. These decays between immunity levels are described by the time constant $1/\Omega$, which is described in the literature to be around 150 days, or a half-life of 100 days^44,45^.

In the model, at the beginning of the pandemic, all individuals from a country start in the susceptible, unvaccinated compartment and then can be infected and moved to one of the infected compartments directly at a specific rate of $\beta _v$, or after being vaccinated at a reduced rate. Individuals who are vaccinated will first move into the susceptible, first dose compartment at a rate of $\alpha _1$ and then into the susceptible, fully vaccinated compartment at a rate of $\alpha _2$. Unlike the conventional SIRDV models, we do not have separate compartments for susceptible vaccinated individuals and insusceptible vaccinated individuals. Instead, our stacked compartment model assumes that each vaccinated individual has some risk of getting infected due to break-through cases^46^. In reality, people who are vaccinated span a spectrum of susceptibility to the virus^47^. Individuals move from the susceptible compartments into the corresponding infected compartment at the unique rates of $\beta _v$ times the unique $\eta _v^{(i)}$ depending on the vaccination-induced immunity level (*i*), and variant, *v*, which accounts for the protection acquired by having the vaccine against a certain variant. From each of the infected compartments, individuals will either move into the diseased compartment at a fixed rate of $\mu _v$, or to the corresponding recovered compartment at a fixed rate of $\gamma _v$. After natural decay from the recovered to the waning recovered compartment, there will be a flow from waning recovered into each of the infected variants’ compartments, except for that of the corresponding variant. This model for reinfection is based on two assumptions that are backed by empirical observations: (i) for some time after infection, there is little chance for reinfection^48^; therefore, there is no flow from recovered to infected, and (ii) after natural decay of infection-induced immunity, chances of reinfection with the same variant remain low^48^; thus there is no flow from waning recovered to infected of the same variant.

Since many individuals are not tested for COVID-19 and asymptomatic cases remain prevalent, there are many unreported cases, especially in countries where testing is less prevalent^49^. Here we predict the actual number of cases by multiplying the reported number of cases by a factor M. The number of cases underreporting/scaling factor M is estimated through data obtained from the serological tests that were conducted in each country^50–57^ to count the number of individuals with immunoglobulin G antibodies against COVID-19. The case underreporting factor, M (actual cases/reported cases), was assumed to be inversely proportional to the number of tests reported. For this study, the underreporting factor was estimated by a population-specific constant divided by the number of tests reported on a particular day. The population-specific constants were estimated from serological testing obtained before widespread vaccination to avoid distortion in the serological testing due to vaccination-induced immunity.

Five countries around the globe representing vastly different geographical and socio-economics were selected for our model-based study. The model was implemented using MATLAB to numerically solve the complete modeling framework. Several model parameters were fitted to these 5 countries, which included *Germany, India, Japan, South Africa, and the United States*. $\gamma$, $\beta _0$, *k*, $\Omega$, and $\mu$ are all variant-specific constants, of which $\gamma$, $\Omega$, and $\mu$ were assumed to be consistent between variants. $\eta _1$ and $\eta _2$ were assumed to be constant for each vaccine type and variant combination. $\alpha _1$, $\alpha _2$, and $\alpha _B$ were determined from *Our World In Data*^58^ and we used the time-varying reported vaccination rates to fit the model to each country’s case history. The prevalence of each variant over time was estimated from the reported sequence prevalence data of each country from GISAID^59–61^. To simplify our simulations, new variants were only considered if they reported at least 15% prevalence of all variants at their peak. All other variants were combined into the compartment referred to as the original strain. $\beta _v$ estimates provide time-dependent transmission rates for each variant; $\beta _v$ was determined by estimating $d_I$ and $d_V$ as demonstrated in (2), while the $\beta _v$ of emerging variants were calculated in the same way, but were scaled using a variant-specific factor that was fit to each variant from the prevalence data. As done in previous work^25^, we introduced several behavioral regions for fitting each country’s time-series data, representing discrete times of pronounced behavioral change where $d_I$ changes value. However, the sense of safety ($d_V$) was assumed to be constant over the simulated region due to the much lower rate of change in the vaccinated population compared to the infected population. With these fixed values in place and using population data^62^ to convert reported cases to a percent of the total population, a gradient-free optimization algorithm in MATLAB was used to optimize the parameters to fit our model to existing variant-specific case data^63^. The root mean square error between our model prediction and the reported data was minimized.

## Results and discussion

### Model fit to country-specific daily cases and interpretation

**Figure 2:**
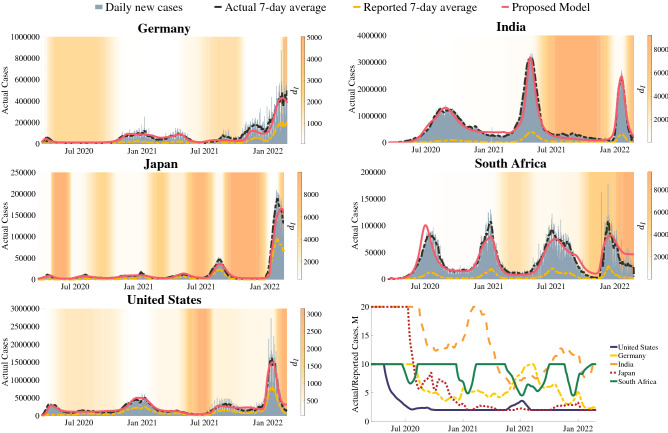
The results of the proposed model fit to Germany, India, Japan, South Africa, and the United States COVID-19 new cases. The model predictions (red, solid) are plotted over the reported 7-day average cases (gold, dashed), estimated actual 7-day average cases (black, dashed), and estimated actual daily cases (gray bars). The level of caution, $d_I$, values from the model fit are plotted as a white-to-orange vertical gradient over time in the plot background, where the color shades of vertical bars correspond to the estimated level of caution. The bottom right plot shows the case underreporting factor, M, for each of the five countries over the course of the simulation. This factor is multiplied by the reported cases to estimate actual cases.

In Fig. 2, we show the model fit to the infection histories of Germany, India, Japan, South Africa, and the United States since March of 2020. These populations were selected to represent a wide range of geographic regions, behavioral and governmental responses to the pandemic, and variant makeups. Solid color vertical stripes in the background of the plots represent behavioral regions in which the population’s level of caution was relatively constant, with the gradients between them representing a smooth transition between behavioral regions. In these regions, the value of $d_I$ changes from a previous to a new value over a set period. Within the transition period, the value of $d_I$ varies according to cosine interpolation between its set starting and ending values, as described by Eq. (3):

3 $$\begin{aligned} d_{I}(t^*) = d_{I, 1} \frac{1+\cos (\varepsilon \pi )}{2} + d_{I, 2} \frac{1-\cos (\varepsilon \pi )}{2}, \;\;\; \varepsilon =\frac{t^* - t_1}{t_2 - t_1}, \end{aligned}$$

where $d_{I, 1}$ and $d_{I, 2}$ are the values of $d_I$ in adjacent behavioral regions, $t_1$ and $t_2$ represent the end and beginning time of these behavioral regions, and $t^*$ and $d_{I, t^*}$ represent the time and value of $d_I$ at an intermediate value between $t_1$ and $t_2$, respectively.

In the two years that we modeled in these countries, significant behavioral shifts in their response to the pandemic occurred about every 3 months. Due to limited available testing, asymptomatic cases, and other societal reasons, the reported number of cases tend to significantly underestimate the actual number of cases in a population. To account for this, we scaled each population’s reported cases by a scaling (underreporting) factor, M. This factor is inversely proportional to the number of tests reported each day and fit for each country based on serological testing. As shown in Fig. 2 for each of the modeled countries, this scaling factor was estimated to range from about 20 actual cases per reported case to about 2. It was assumed that M would never be below 2 due to an estimated 40.5% of confirmed COVID-positive individuals being asymptomatic^64^.

### Estimating dynamic population immunity

**Figure 3:**
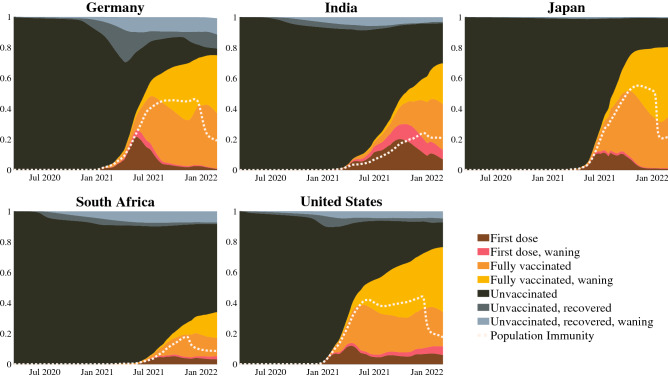
Proportions of various immunity levels in five countries modeled over time. Overlain in the white, dotted line measures population immunity, weighted proportionally to all population groups and prevalent variants. A value of zero represents no population immunity, and a value of one represents complete population immunity and no susceptibility to future infections.

Valuable insights into a population’s state in a pandemic can be drawn from the distribution among the compartments and immunity levels in the model. To illustrate population immunity throughout the pandemic, the proportions of the population in each vaccination level, as well as those who are unvaccinated and previously infected, are plotted in Fig. 3. As access to vaccines increases in each country, the proportion of individuals without immunity decays. This visualization of population immunity also illustrates the effects of waning immunity, both for vaccine-induced and infection-induced immunity, predicting that much of the immunized population is waning toward the lower limit of their immunity against infection. This emphasizes the importance of booster vaccinations to maintain immunity and prevent further infection.

The information on the immunity levels and distribution among compartments is used to estimate the average population immunity over time. This is a sum of the immunity of each compartment towards infection, weighted by the distribution of the population amongst these compartments and the prevalence of each of the disease variants. For both vaccination-induced and infection-induced immunity, a significant waning of immunity causes the population’s aggregate immunity to decay towards the lower waning levels, requiring regular vaccination to maintain population immunity. Before the advent and distribution of vaccines, each population’s average immunity was around zero, indicating complete susceptibility. Through the widespread distribution of the vaccines, average immunity rose fairly consistently in each modeled country until late 2021, when the Omicron variant emerged with reduced protection from the overall vaccines and an increased risk of reinfection. This correlates directly to severe peaks in cases globally. By combining the effects of vaccine-induced immunity, infection-induced immunity, waning immunity, reinfection risks, and variant prevalence, the aggregate immunity estimate presented in this paper provides a valuable metric for assessing a population’s susceptibility to further infection.

### Variant dynamics and sensitivity

**Figure 4:**
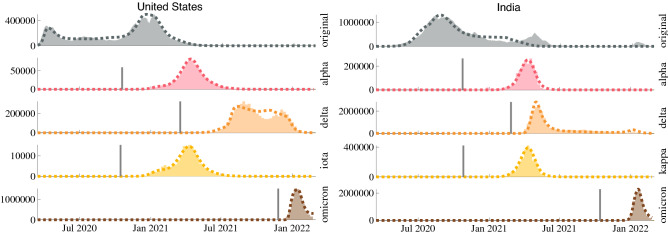
Results of the model fit to each of the dominant variants in the United States and India (dotted), plotted against the estimated actual daily cases (bar). The transmission start date of each variant is indicated by a vertical black bar.

**Figure 5:**
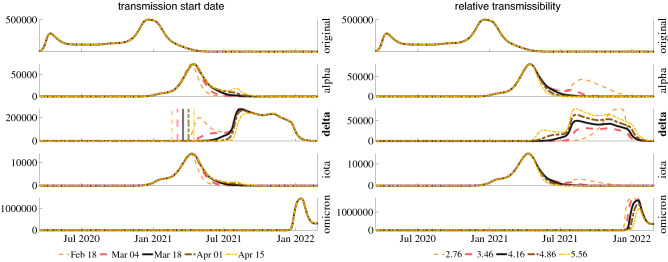
Effect of transmission start date and relative transmissibility of the Delta variant on COVID-19 cases among all dominant variants in the United States.

The infection histories of each of the dominant variants in the United States and India are shown in Fig. 4, along with the model’s ability to reproduce these curves. We selected the United States and India as examples from developed and developing countries. By fitting the model parameters to the observed variant curves, we can deduce helpful insight into the relevant disease characteristics of each variant. For example, in the United States, our model estimates that the Alpha, Delta, and Omicron variants are about 1.8, 4.2, and 21.8 times more transmissible than the original strain of COVID-19. In India, a similar analysis estimates the Alpha, Delta, and Omicron variants to be around 2.0, 5.5, and 11.8 times more transmissible than the original strain. It is important to note that transmissibility is interrelated with vaccine efficacy and infectious period in our model equations. The net population transmissibility estimates are based on reported values for vaccine efficacy against each variant and an infectious period of ten days assumed on an average for all the variants. In addition to estimating transmissibility, the proposed model can estimate the date of the initial spread of each variant in a population. The model’s estimate is more specific than estimates such as the first reported case of a variant because it is likely that either many instances of a particular variant were not identified, resulting in an estimate later than the actual first case, or the first identified instance was well contained and did not lead to further spread, resulting in an estimate before the real beginning of the variant spread. Figure 5 explores a sensitivity analysis of the transmission start date and relative transmissibility of the Delta variant, revealing that the model is quite sensitive to small changes in these parameters. This sensitivity further supports our estimates of these variant parameters.

By conducting simulations using the model with a range of parameters for a single variant and population, we can estimate the sensitivity of our simulation results to perturbations in the variant parameters. Figure 5 shows a sensitivity analysis of the transmission start date and relative transmissibility ($\beta _v/\beta _{original}$). Transmission start date, as described above, is the date used in the model fit to predict the first case that led to widespread transmission, rather than the actual first case or first reported case of a variant, as described in the previous paragraph. The relative transmissibility of a variant is the ratio of the estimated transmissibility of the variant to the estimated transmissibility of the original strain of the virus. The delta variant was chosen to perform the sensitivity analysis because it reached nearly complete dominance over other variants at its peak, and its spread occurred before and after other preeminent variants. With this analysis of all key variants, a clear competitive interaction between the prevalent variants is observed. As the transmissibility and infectious period of a particular variant was increased, or it was introduced at an earlier date, its infections increased, and the infections of all other variants that occur following its initial spread decreased. Notably, a similar sensitivity analysis on the infectious period of a particular variant shows the same trends as relative transmissibility, supporting a metric such as $R_0$ that combines transmissibility and infectious period to describe the nature of a variant.

### Vaccine allocation study

**Figure 6:**
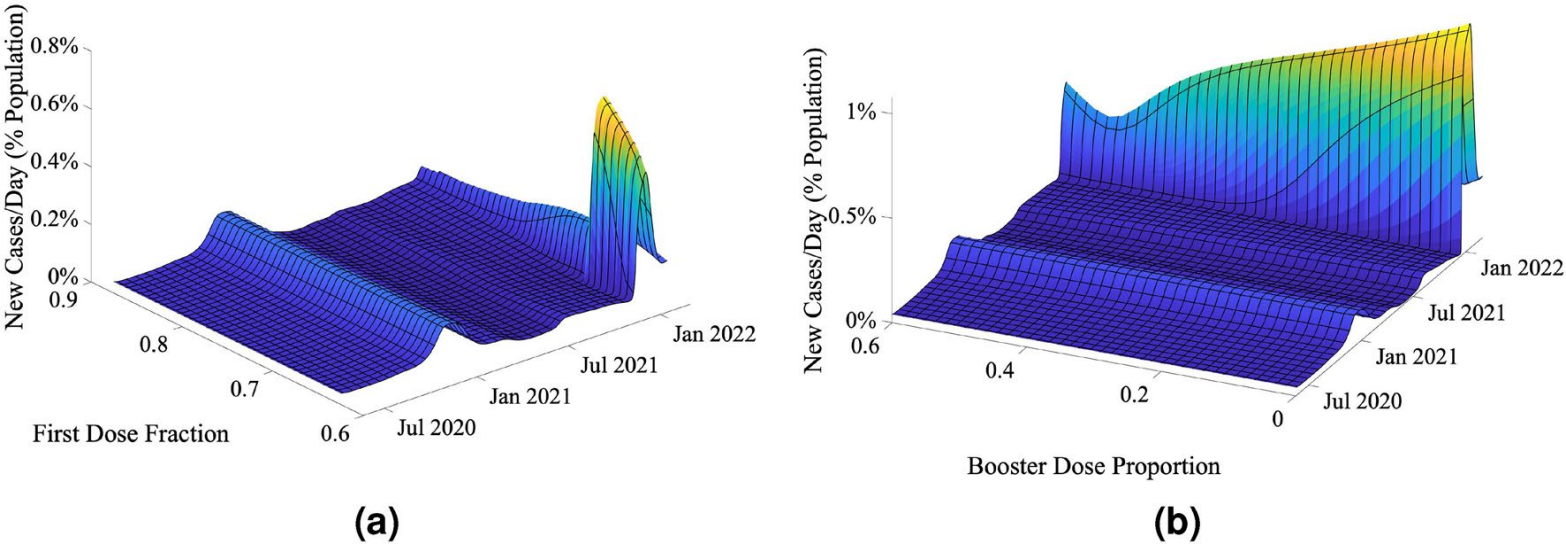
Evaluation of vaccine allocation strategies, assuming that only a set number of the first, second, and booster vaccine doses combined can be administered ($\alpha _{1,prop}+\alpha _{2,prop}+\alpha _{B,prop}=1$). Sensitivity of pandemic trajectories with respect to (**a**) a fixed number of doses for the booster vaccines (60% of the total doses being administered), and the relative proportions of first and second vaccine doses being varied, and (**b**) a variable number of booster doses, where changes in the amount of booster doses were accommodated by changing the proportions of the first and second doses equally.

Given the model’s ability to predict pandemic trends concerning different variants, we investigated its ability to draw conclusions for differing vaccine distribution rates using the United States as an example case. Figure 6 shows several contemplative/retrospective studies performed using the model. To perform these simulations, at each time point, the total number of vaccine doses available was determined from past vaccine data, and these vaccines were redistributed among the first, second, and booster doses, given a certain proportion of each. While the first and second doses of the vaccine in question were imposed at the first recorded date of vaccination in the US (December 14, 2020), the booster dose was started much later (August 17, 2021) when the booster doses became available. Thus, before the advent of the booster dose, vaccines were just distributed between the first and second vaccine doses. In addition, a parameter was added to account for the fact that a certain proportion of the population will refuse to take any vaccine dose. A threshold value was placed on the layer within the stacked model representing unvaccinated individuals to set $\alpha _1$ to 0 if the proportion of the population in this group dropped below the threshold. The same value was used to threshold the layers of the stacked model containing individuals with the first dose of the vaccine (i.e., first dose and first dose, waning). If the total individuals in these compartments dropped below this threshold, $\alpha _2$ was set to 0. For this part of the trend study, the population that does not take vaccination was assumed to be 10% of the total population.

In Fig. 6a, dose allocation between the first and second doses of the vaccine is examined. The pandemic trajectories over time are plotted for different values of the fraction of the first dose, defined in Eq. (4) as

4 $$\begin{aligned} x_{first \,dose} = \frac{\alpha _{1,prop}}{\alpha _{1,prop} + \alpha _{2,prop}}, \end{aligned}$$

where $\alpha _{1,prop}$ and $\alpha _{2,prop}$ are the proportions of the total number of vaccine doses at each time point allocated to the first and second vaccine doses, respectively. In Fig. 6a, the pandemic trajectories are relatively stable for large values of $x_{firstdose}$ close to 1. However, the peak in infectious cases around January 2022 grows rapidly for $x_{firstdose} \approx 0.67$. This indicates that when the first dose of the vaccine is prioritized significantly over the second dose (by a ratio of approximately 2:1), the pandemic trajectories tend to be relatively stable and do not significantly vary when the dose prioritization is changed a small amount. However, when the first dose becomes less prioritized, there is a critical threshold below which infectious cases can proliferate significantly.

Figure 6b shows the results of the booster vaccine dose being varied in relation to the other doses. The proportions of the first, second, and booster doses, $\alpha _{1,prop}$, $\alpha _{2,prop}$, and $\alpha _{B,prop}$ respectively, were initially set to 0.25, 0.15, and 0.60. The sum of the proportions of the three doses was always set to be 1. The proportion of the booster dose was varied, and each change in proportion was reflected by changing the proportions of the first and second dose proportions in equal amounts. From Fig. 6b, it is clear that as booster doses increase, the peak in infectious cases around January 2022 begins to decrease until a booster dose proportion of approximately 0.5, where the number of infectious cases begins to increase. This could happen due to insufficient numbers of individuals being given the base levels of immunity from the first and second doses, with too much prioritization given to individuals who had already been vaccinated twice, leading to a proliferation in infectious cases.

## Closing remarks

The proposed modeling method can recreate the history of the COVID-19 pandemic up to the spring of 2022. It can estimate future case trends accounting for variants, waning immunity, population behavior, and vaccine roll-out strategies. This infectious disease dynamic modeling framework can be used for future population disease studies to study the effects of several critical aspects of the pandemic, such as behavioral mandates, the advent and interaction of variants, and the dispersion of multi-dose vaccines with waning immunity. In this paper, we propose methods of quantifying various population-level metrics that are useful in the analysis of a pandemic, such as the relative strength of variants, the aggregate immunity of a population, and the effectiveness of various vaccination rollout strategies. We have included a few examples that provide insight into the competitive nature of disease variants and the outcomes of a range of vaccine allocation strategies. As with any mathematical model, several limitations exist. While the level of caution and sense of safety are accounted for, the model does not fully account for complex, locally heterogeneous social structures. We assume homogeneous mixing of populations and a fixed population level that does not reckon large-scale emigration and immigration of individuals. As travel resumes and if conflicts or economic opportunity-drive mass migration, ignoring the movement of populations between countries may limit the compartmental model’s ability to predict future cases accurately. Heterogeneity in the susceptibility of individuals within a population can have a significant effect on the infection trajectory, so further studies can investigate how heterogeneity based models^65^ interact with the model presented here. Another potential limitation of the proposed model is how we account for the underreporting of cases. Since we do not have serological data that can provide insight into the under-reporting of cases after the introduction of the vaccine, the ability to estimate the actual number of infected cases reduces for these circumstances. The model’s predictive capabilities will be limited by the uncertainties associated with evolving COVID-19. Some of the infection, variants and population specific estimated COVID parameters may get adjusted over time as new information becomes available. The model and its framework have the capability to incorporate new and more accurate data as they become available. With correct model parameter inputs, it is one of the first models that allow studying coupled effects of vaccine roll-out strategies, vaccine efficacies, VOCs, waning immunity, population level of caution, sense of safety, and the under-reporting of cases to predict potential infectious disease trends.

## Data Availability

The datasets analyzed during the current study are currently available in the following repositories. COVID case data were obtained from the Center for Systems Science and Engineering (CSSE) COVID-19 Data Repository at Johns Hopkins University^62^, https://github.com/CSSEGISandData/COVID-19. Country-specific prevalence of each variant was obtained from the GISAID $EpiCov^{TM}$ database^59–61^, https://www.gisaid.org. Daily vaccinations from each country were provided by Our World In Data^66^, https://ourworldindata.org/coronavirus.

## Code availability

The MATLAB source code for the model is available on GitHub, https://github.com/zlajoie/covidmodeling.git.

## References

[1] Johns Hopkins University Coronavirus Resource Center. Accessed 30 Apr 2022. https://coronavirus.jhu.edu/ (Johns Hopkins Coronavirus Resource Center, 2022).

[2] Ke X, Hsiao C (2022). Economic impact of the most drastic lockdown during COVID-19 pandemic—The experience of Hubei, China. J. Appl. Economet..

[3] Chen X, Huang H, Ju J, Sun R, Zhang J (2022). Impact of vaccination on the COVID-19 pandemic in US states. Sci. Rep..

[4] Telenti A (2021). After the pandemic: Perspectives on the future trajectory of COVID-19. Nature.

[5] Sah P (2021). Accelerated vaccine rollout is imperative to mitigate highly transmissible COVID-19 variants. eClinicalMedicine.

[6] Zimmer, C., Corum, J., Wee, S.-L. & Kristoffersen, M. Coronavirus vaccine tracker. *The New York Times*. https://www.nytimes.com/interactive/2020/science/coronavirus-vaccine-tracker.html (2020).

[7] Ritchie, H. *et al.* Coronavirus pandemic (covid-19). In *Our World in Data*. Accessed 30 Apr 2022. https://ourworldindata.org/covid-vaccinations (2020).

[8] CDC. *Coronavirus Disease 2019 (COVID-19)*. Accessed 30 Apr 2022. https://www.cdc.gov/coronavirus/2019-ncov/variants/variant-classifications.html (Centers for Disease Control and Prevention, 2022).

[9] Nasreen S (2021). Effectiveness of COVID-19 vaccines against variants of concern in Ontario, Canada. MedRxiv..

[10] Lustig Y (2021). Eurosurveillance | neutralising capacity against delta (b.1.617.2) and other variants of concern following comirnaty (BNT162b2, BioNTech/pfizer) vaccination in health care workers, Israel. Eurosurveillance.

[11] Katella, K. *Omicron, Delta, Alpha, and More: What to Know About the Coronavirus Variants*. Accessed 30 Apr 2022. https://www.yalemedicine.org/news/covid-19-variants-of-concern-omicron (Yale Medicine, 2022).

[12] Bernal J, Andrews N, Gower C, Gallagher E (2021). Effectiveness of covid-19 vaccines against the b.1.617.2 (delta) variant. N. Engl. J. Med..

[13] Cherian S (2021). Convergent evolution of SARS-CoV-2 spike mutations, l452r, e484q and p681r, in the second wave of COVID-19 in Maharashtra, India. BioRxiv..

[14] Takashita E (2022). Efficacy of antibodies and antiviral drugs against covid-19 omicron variant. N. Engl. J. Med..

[15] Matrajt L (2021). Optimizing vaccine allocation for COVID-19 vaccines shows the potential role of single-dose vaccination. Nat. Commun..

[16] Bar-On YM (2021). Protection of BNT162b2 vaccine booster against covid-19 in Israel. N. Engl. J. Med..

[17] Kumar VM, Pandi-Perumal SR, Trakht I, Thyagarajan SP (2021). Strategy for COVID-19 vaccination in India: The country with the second highest population and number of cases. NPJ Vaccines.

[18] Ferreras E (2018). Single-dose cholera vaccine in response to an outbreak in Zambia. N. Engl. J. Med..

[19] Casey R (2019). Immunogenicity of fractional-dose vaccine during a yellow fever outbreak—Final report. N. Engl. J. Med..

[20] *The Pfizer BioNTech (BNT162b2) COVID-19 Vaccine: What You Need to Know*. Accessed 30 Apr 2022. https://www.who.int/news-room/feature-stories/detail/who-can-take-the-pfizer-biontech-covid-19-vaccine-what-you-need-to-know. (World Health Organization, 2022).

[21] Balch, B. *Canada Took a Risk Delaying Second COVID-19 Vaccine Doses. Now, Its Vaccination Campaign is One of the Best in the World*. https://www.aamc.org/news-insights/canada-took-risk-delaying-second-covid-19-vaccine-doses-now-its-vaccination-campaign-one-best-world (Association of American Medical Colleges, 2021).

[22] Zimmer, C. To speed vaccination, some call for delaying second shots. *The New York Times*. Accessed 30 Apr 2022. https://www.nytimes.com/2021/04/09/health/covid-vaccine-second-dose-delay.html (2021).

[23] Ujjainia R (2021). Effect monitoring and insights from vaccination program of healthcare workforce from a tertiary level hospital in India against SARS-CoV-2. MedRxiv..

[24] Chemaitelly H, Tang P, Hasan MR, Almukdad EA (2021). Waning of bnt162b2 vaccine protection against sars-cov-2 infection in Qatar. N. Engl. J. Med..

[25] Usherwood T, LaJoie Z, Srivastava V (2021). A model and predictions for COVID-19 considering population behavior and vaccination. Sci. Rep..

[26] Steinegger B, Arola-Fernández L, Granell C, Gómez-Gardeñes J, Arenas A (2022). Behavioural response to heterogeneous severity of covid-19 explains temporal variation of cases among different age groups. Philos. Trans. R. Soc. A.

[27] Roda WC, Varughese MB, Han D, Li MY (2020). Why is it difficult to accurately predict the COVID-19 epidemic?. Infect. Dis. Model..

[28] Liu M, Thomadsen R, Yao S (2020). Forecasting the spread of COVID-19 under different reopening strategies. Sci. Rep..

[29] Ram V, Schaposnik LP (2021). A modified age-structured sir model for covid-19 type viruses. Sci. Rep..

[30] Bubar KM (2021). Model-informed covid-19 vaccine prioritization strategies by age and serostatus. Science.

[31] Kim D, Keskinocak P, Pekgün P, Yildirim İ (2022). The balancing role of distribution speed against varying efficacy levels of covid-19 vaccines under variants. Sci. Rep..

[32] Postnikov EB (2020). Estimation of COVID-19 dynamics on a back-of-envelope: Does the simplest SIR model provide quantitative parameters and predictions?. Chaos Solitons Fractals.

[33] Brauer F (2017). Mathematical epidemiology: Past, present, and future. Infect. Dis. Model..

[34] Kermack WO, McKendrick AG, Walker GT (1927). A contribution to the mathematical theory of epidemics. Proc. R. Soc. Lond. Ser. A.

[35] Mak, H.-Y., Dai, T. & Tang, C. S. Managing two-dose COVID-19 vaccine rollouts with limited supply: Operations strategies for distributing time-sensitive resources. In *SSRN Scholarly Paper 3790836, Social Science Research Network*. Accessed 30 Apr 2022. https://papers.ssrn.com/abstract=3790836 (2022). 10.1111/poms.13862 PMC9538244 36246547

[36] García YE (2022). Projecting the impact of covid-19 variants and vaccination strategies in disease transmission using a multilayer network model in Costa Rica. Sci. Rep..

[37] Gonzalez-Parra G, Martínez-Rodríguez D, Villanueva-Micó R-J (2021). Impact of a new SARS-CoV-2 variant on the population: A mathematical modeling approach. MedRxiv..

[38] Lipsitch M, Dean NE (2020). Understanding covid-19 vaccine efficacy. Science.

[39] Read JM, Bridgen JRE, Cummings DAT, Ho A, Jewell CP (2021). Novel coronavirus 2019-ncov (covid-19): Early estimation of epidemiological parameters and epidemic size estimates. Philos. Trans. R. Soc. B Biol. Sci..

[40] Faucher B (2022). Agent-based modelling of reactive vaccination of workplaces and schools against covid-19. Nat. Commun..

[41] Liu Q-H (2022). Model-based evaluation of alternative reactive class closure strategies against covid-19. Nat. Commun..

[42] Gleeson JP (2022). Calibrating covid-19 susceptible-exposed-infected-removed models with time-varying effective contact rates. Philos. Trans. R. Soc. A Math. Phys. Eng. Sci..

[43] Lau H (2021). Evaluating the massive underreporting and undertesting of COVID-19 cases in multiple global epicenters. Pulmonology.

[44] Goldberg Y (2021). Waning immunity after the BNT162b2 vaccine in Israel. N. Engl. J. Med..

[45] Levin E, Lustig Y, Cohen C, Fluss R (2021). Waning immune humoral response to BNT162b2 covid-19 vaccine over 6 months. N. Engl. J. Med..

[46] Birhane M (2021). COVID-19 vaccine breakthrough infections reported to CDC—United States, January 1–April 30, 2021. Morb. Mortal. Wkly. Rep..

[47] Jeyanathan M (2020). Immunological considerations for COVID-19 vaccine strategies. Nat. Rev. Immunol..

[48] Altarawneh HN (2022). Protection against the omicron variant from previous SARS-CoV-2 infection. N. Engl. J. Med..

[49] Biswas RK, Afiaz A, Huq S (2020). Underreporting COVID-19: The curious case of the Indian subcontinent. Epidemiol. Infect..

[50] Yamamoto S (2021). Seroprevalence of SARS-CoV-2 antibodies in a national hospital and affiliated facility after the second epidemic wave of Japan. J. Infect..

[51] Nawa N (2020). Seroprevalence of SARS-CoV-2 IgG antibodies in Utsunomiya city, greater Tokyo, after first pandemic in 2020 (u-CORONA): A household- and population-based study. MedRxiv..

[52] Silveira MF (2020). Population-based surveys of antibodies against SARS-CoV-2 in Southern Brazil. Nat. Med..

[53] Song S-K (2020). IgG seroprevalence of COVID-19 among individuals without a history of the coronavirus disease infection in Daegu, Korea. J. Korean Med. Sci..

[54] Nah E-H, Cho S, Park H, Hwang I, Cho H-I (2021). Nationwide seroprevalence of antibodies to SARS-CoV-2 in asymptomatic population in South Korea: A cross-sectional study. BMJ Open.

[55] Stout R, Rigatti S (2021). Seroprevalence of SARS-CoV-2 antibodies in the US adult asymptomatic population as of September 30, 2020 | infectious diseases | JAMA network open | JAMA network. JAMA Netw. Open.

[56] Murhekar MV (2020). Prevalence of SARS-CoV-2 infection in India: Findings from the national serosurvey, May–June 2020. Indian J. Med. Res..

[57] Harries M (2021). SARS-CoV-2 seroprevalence in Germany—A population based sequential study in five regions. MedRxiv..

[58] Mathieu E (2021). A global database of COVID-19 vaccinations. Nat. Hum. Behav..

[59] Khare S (2021). GISAID’s role in pandemic response. China CDC Wkly..

[60] Elbe S, Buckland-Merrett G (2017). Data, disease and diplomacy: GISAID’s innovative contribution to global health. Glob. Challenges (Hoboken, NJ).

[61] Shu Y, McCauley J (2017). GISAID: Global initiative on sharing all influenza data—From vision to reality. Eurosurveillance.

[62] Dong E, Du H, Gardner L (2020). An interactive web-based dashboard to track COVID-19 in real time. Lancet Infect. Dis..

[63] *Bound Constrained Optimization Using Fminsearch; Fminsearchbnd, Fminsearchcon*. https://www.mathworks.com/matlabcentral/fileexchange/8277-fminsearchbnd-fminsearchcon (MathWorks, 2022).

[64] Ma Q, Liu J, Liu Q (2021). Global percentage of asymptomatic SARS-CoV-2 infections among the tested population and individuals with confirmed COVID-19 diagnosis: A systematic review and meta-analysis | global health | JAMA network open | JAMA network. JAMA Netw. Open.

[65] Rose C (2021). Heterogeneity in susceptibility dictates the order of epidemic models. J. Theor. Biol..

[66] Ritchie, H. *et al.* Coronavirus pandemic (COVID-19). In *Our World in Data*. https://ourworldindata.org/coronavirus (2020).

